# Venereal Sores—Iodoform

**Published:** 1878-09

**Authors:** 


Venereal Sores—Iodoform.—Locally, iodoform, as a dry powder, brushed lightly over the surface with a moistened camel-hair pencil, has been for three years my almost invariable treatment for venereal sores, especially the local chancre. During the last few months I have often substituted for the dry powder an etherial solution (one part of iodoform in six or eight of ether). The sore is touched or dabbed with a pencil dipped in the etherial solution, according to its size and depth, lightly or copiously. The ether quickly evaporates, leaving a thin pellicle of iodoform, that as effectually stays the spread and produces healing of chancres as does the more copiously applied dry powder. Thus the surface is covered more exactly, and the disagreeable smell of iodoform is too faint to attract attention. The sore is well washed with water and dried before the iodoform is applied, and the surface is lastly protected by a bit of dry lint. When the secretion is abundant, the dressing must be renewed twice daily, but in three or four days the amount of discharge becomes so scant that one dressing *per diem* suffices. In this way venereal sores heal quickly. Pain subsides at once; the sore is well in a week or ten days, and the chances of consecutive inoculation or bubo are greatly lessened. In a very few cases the application of iodoform gives momentary smarting, which is very bearable; even the ethereal solution does not hurt, and usually the patient declares the application to be quite painless. I avoid using iodoform on inflamed sores, or on simple granulating wounds; but indolent non-specific ulcers are rapidly improved by iodoform locally applied. (Mr. Berkly Hill, p. 180.)—*Braithwaite's Retrospect of Practical Med. and Surg.*

